# Characterization of the genetic variation present in *CYP3A4* in three South African populations

**DOI:** 10.3389/fgene.2013.00017

**Published:** 2013-02-18

**Authors:** Britt Drögemöller, Marieth Plummer, Lundi Korkie, Gloudi Agenbag, Anke Dunaiski, Dana Niehaus, Liezl Koen, Stefan Gebhardt, Nicol Schneider, Antonel Olckers, Galen Wright, Louise Warnich

**Affiliations:** ^1^Department of Genetics, Stellenbosch UniversityStellenbosch, South Africa; ^2^Central DNA Sequencing Facility, Stellenbosch UniversityStellenbosch, South Africa; ^3^Department of Psychiatry, Stellenbosch UniversityTygerberg, South Africa; ^4^Department of Obstetrics and Gynaecology, Stellenbosh UniversityTygerberg, South Africa; ^5^DNAbiotec (Pty) Ltd. and Faculty of Health Sciences, University of PretoriaPretoria, South Africa

**Keywords:** *CYP3A4*, pharmacogenetics, South African populations, Xhosa, mixed ancestry, Khoisan

## Abstract

The CYP3A4 enzyme is the most abundant human cytochrome P450 (CYP) and is regarded as the most important enzyme involved in drug metabolism. Inter-individual and inter-population variability in gene expression and enzyme activity are thought to be influenced, in part, by genetic variation. Although Southern African individuals have been shown to exhibit the highest levels of genetic diversity, they have been under-represented in pharmacogenetic research to date. Therefore, the aim of this study was to identify genetic variation within *CYP3A4* in three South African population groups comprising of 29 Khoisan, 65 Xhosa and 65 Mixed Ancestry (MA) individuals. To identify known and novel *CYP3A4* variants, 15 individuals were randomly selected from each of the population groups for bi-directional Sanger sequencing of ~600 bp of the 5′-upstream region and all thirteen exons including flanking intronic regions. Genetic variants detected were genotyped in the rest of the cohort. In total, 24 SNPs were detected, including CYP3A4^*^12, CYP3A4^*^15, and the reportedly functional CYP3A4^*^1B promoter polymorphism, as well as two novel non-synonymous variants. These putatively functional variants, p.R162W and p.Q200H, were present in two of the three populations and all three populations, respectively, and *in silico* analysis predicted that the former would damage the protein product. Furthermore, the three populations were shown to exhibit distinct genetic profiles. These results confirm that South African populations show unique patterns of variation in the genes encoding xenobiotic metabolizing enzymes. This research suggests that population-specific genetic profiles for *CYP3A4* and other drug metabolizing genes would be essential to make full use of pharmacogenetics in Southern Africa. Further investigation is needed to determine if the identified genetic variants influence CYP3A4 metabolism phenotype in these populations.

## Introduction

The human CYP3A enzymes are regarded as the most prominent Cytochrome P450 (CYP) subfamily in facilitating the elimination of drugs, other xenobiotic compounds and endogenous molecules from the body (Lamba et al., 2002). The pharmacogenetically relevant CYP3A4 is responsible for metabolizing 50–60% of all clinically prescribed drugs (Guengerich, 1999) and is listed among The Pharmacogenetics and Pharmacogenomics Knowledge Base's (PharmGKB's) “very important pharmacogenes” (http://www.pharmgkb.org/gene/PA130?tabType=tabVip). CYP3A4 can be inhibited by drugs (e.g., ketoconazole and ritonavir) and is often involved in unfavorable drug-drug interactions, due to its ability to accommodate two or more similar or dissimilar molecules in its active site (Sevrioukova and Poulos, 2013). The enzyme is predominantly expressed in the liver and small intestine (Shimada and Guengerich, 1989). Expression has as much as 40-fold variation between individual human livers and a 10-fold variation in the metabolism of CYP3A4 substrates *in vivo* (Shimada and Guengerich, 1989; Lown et al., 1995; Guengerich, 1999). While complex regulatory pathways and environmental factors are important, it is suspected that a portion of this inter-individual variation can be attributed to genetic variants located within the coding gene regions as well as its core regulatory regions, which affect either the expression level or the functional protein of the gene (Steimer and Potter, 2002; Lamba et al., 2002).

Few pharmacogenetically-relevant polymorphisms have been identified in the *CYP3A4* gene; however, some polymorphisms have been associated with, amongst others, immunosuppressant dose requirements (Elens et al., 2011), clopidogrel response variability (Angiolillo et al., 2006), and withdrawal symptoms and adverse reactions in patients receiving methadone treatment (Chen et al., 2011). Furthermore, a rare haplotype, CYP3A4^*^20, results in a complete loss of function (Westlind-Johnsson et al., 2006), while CYP3A4^*^1B is suspected to alter the expression levels of CYP3A4 (Westlind et al., 1999), although conflicting results have been reported (Wang et al., 2011). Although genetic variants in the *CYP3A4* gene have been extensively studied in populations such as Caucasians, Asians, and African-Americans, little research has been conducted in present-day African populations, including those indigenous to South Africa (Warnich et al., 2011). Not only are these research disparities observed in candidate gene studies, but they also extend to recent large scale re-sequencing projects such as the 1000 Genomes Project, which although comprehensively examining the genomic variation present in many individuals, provides no information for South African populations (1000 Genomes Consortium, 2010).

We have therefore tried to aid in addressing the disparity of pharmacogenetic data that exists for South African populations by analyzing three of the diverse population groups, which are representative of: (1) the most ancient population group: the Khoisan, (2) the most globally-admixed population group: the South African Mixed Ancestry (MA) population, and (3) the largest language family in South Africa: the Bantu-speaking population group, represented by the Xhosa population. The ancient Khoisan population used in this study consisted of individuals from the !Kung and Khwe linguistic groups (Chen et al., 2000). These individuals are descendant from people of the latter Stone Age and are believed to be some of the first lineages of *Homo sapiens* (Kaessmann and Pääbo, 2002). The MA population, with Xhosa, Khoisan, European, and Asian ancestral contributions, has been shown to exhibit the highest levels of admixture across the globe (Tishkoff et al., 2009) and is therefore of interest for pharmacogenetic applications as genetic variants present in many different populations may affect these individuals as has been reported for other admixed populations such as those from Brazil (Suarez-Kurtz, 2005, 2010; Suarez-Kurtz et al., 2012). Lastly, 9 of the 11 official South African languages are classified as Bantu languages (Warnich et al., 2011), spoken by ~75% of the total South African population, and therefore it is imperative that representatives of this group are included in pharmacogenetic research. In this study, we utilized the Xhosa population, which are representative of the Nguni-speaking tribes (Warnich et al., 2011) and are the biggest Bantu-speaking population in the Western Cape of South Africa, where this research was conducted.

In our experience, it is important that pharmacogenes, such as the *CYP* genes are comprehensively characterized in South African populations, as we have discovered both novel alleles and unique variation profiles for the *CYP2C19* and *CYP2D6* genes (Drögemöller et al., 2010; Wright et al., 2010). It is hoped that the comprehensive characterization of *CYP3A4* in these populations will aid future *CYP3A4* genotype-phenotype studies in African populations to determine whether functionally relevant *CYP3A4* polymorphisms exist that have an impact on drug metabolism phenotype. We therefore screened the 5′-flanking region and thirteen exonic regions of the *CYP3A4* gene in the three South African populations described above in order to determine which common allelic variants, novel or previously characterized, are present.

## Materials and methods

### Clinical samples

Ethical approval was obtained for this study from the Human Research Ethics Committee of Stellenbosch University (S12/07/190) and informed consent was acquired from all participants. Genomic DNA (gDNA) was available for 29 Khoisan, 65 Xhosa, and 65 MA healthy individuals. The Khoisan samples used in this study were collected from !Kung and Khwe speaking individuals from the Schmidtsdrift region of the Kalahari desert in the Northern Cape Province of South Africa (Chen et al., 2000), while samples from the Xhosa and MA populations were collected from the Western Cape Province of South Africa.

### Polymerase chain reaction (PCR) amplification

Primers were designed to amplify ~600 bp of the 5′-upstream region and all 13 exons with flanking intronic regions of *CYP3A4* (GenBank: AF280107.1; Ensembl ID: ENSG00000160868) (refer to Table 1 for primer sequences). PCR amplifications were carried out in a total reaction volume of 25 μl, with each reaction containing 20–30 ng of gDNA, 10 pmol of each primer, 320 μM dNTPs, 4 mM MgCl_2_, 0.5 U BIOTAQ™ DNA polymerase and 1X Reaction Buffer. All reagents were supplied by Bioline, London, UK. The reaction cycle conditions consisted of an initial denaturation step at 94°C for 3 min, followed by 30 cycles of 15 s denaturation at 94°C, 15 s annealing at varying temperatures (refer to Table 1 for specific annealing temperatures), and 30 s extension at 72°C, with a final extension at 72°C for 5 min.

**Table 1:** **Sequencing Primers and PCR conditions**.

**Region**	**Primer name**	**Sequence (5′-3′)**	***T*_*m*_ (°C)**	**Product size (bp)**
5′-upstream	5′_F	CAG AAG GGA TGA CAT GCA GA	60	767
	5′_R	GGC TAT GTG CAT GGA GCT TT		
Exon 1	E1_F	GAT TCT TTG CCA ACT TCC AAG	60	363
	E1_R	GAT TAG CAC CCC AAG TCC AA		
Exon 2	E2_F	GCA GGA AAG GAC CTG ATG AA	60	323
	E2_R	AAG CTG CTC TTG GCA ATC AT		
Exon 3	E3_F	TGA CGT CTC CAA ATA AGC TTC C	60	301
	E3_R	AGG TTG ACA AGA GCT TCA TCC		
Exon 4	E4_F	AGG ATC AAA GTC TGG CTT CC	60	305
	E4_R	GGA TGA AGT GGA CGT GGA AC		
Exon 5	E5_F	TCT AGC ATA GGG CCC ATC AC	60	352
	E5_R	CA GTG GAC TAC CCC TTG GAA		
Exon 6	E6_F	CCA AGG GGT AGT CCA CTG AA	55	362
	E6_R	GGA ATA ACC CAA CAG CAG GA		
Exon 7	E7_F	TGG AGT GTG ATA GAA GGT GA	55	516
	E7_R	TTG TGA CAG GGG GCT GAT AG		
Exon 8	E8_F	TGC TCC AGG TAA ATT TTG CAC	60	369
	E8_R	CAA ACC CCA CTT TCT GCA TT		
Exon 9	E9_F	CAT CCT GCT TTC CAA GGA	60	418
	E9_R	CCT GCA TGC CTC TAG AAA GTG		
Exon 10	E10_F	TGA TGC CCT ACA TTG ATC TGA	60	391
	E10_R	CTG CCA GTA GCA ACC ATT TG		
Exon 11	E11_F	CCC GAA TGC TTC CCA CCT	60	506
	E11_R	GGC AGA ATA TGC TTG AAC CAG		
Exon 12	E12_F	GAC TGA AAG CTC CTA TAG TGT C	60	598
	E12_R	CCA TGC TAA TCT ACA TGG GCT		
Exon 13	E13_F	GCC ATC ATA CCT AAT AAT CTG G	60 Xhosa	988
	E13_R	AT GTG CAG GAA AGC ATC TGA	55 Others	

5′, 5′-upstream; E, exon; F, forward; R, reverse.

### DNA sequencing

To identify common *CYP3A4* genetic variation occurring in each of the three populations, 15 individuals were randomly selected from every population groups for bi-directional sequence analysis, allowing for detection of alleles with a frequency of more than 10%, with 95% confidence. The PCR products from each of the 13 amplicons were purified with SureClean (Bioline) and bi-directionally sequenced using the BigDye® Terminator v3.1 Cycle Sequencing Kit (Applied Biosystems, CA, USA), after which capillary electrophoresis was performed by the Central Analytical Facility of Stellenbosch University on a 3130*xl* Genetic Analyser (Applied Biosystems). The obtained sequences were subsequently compared to the reference sequence (GenBank: AF280107.1; Ensembl ID: ENSG00000160868) to detect the presence of variants. The generated sequence data also served to ensure that the reaction conditions used did not amplify any of the associated *CYP3A4* isoforms or pseudogenes. The effect of the detected variants was determined using the software programs Sorting Intolerant From Tolerant (SIFT), Polymorphism Phenotyping (PolyPhen) and the Alternative Splice Site Predictor (ASSP) (http://www.es.embnet.org/~mwang/assp.html; Ramensky et al., 2002; Ng and Henikoff, 2003).

### Genotyping of the detected variants

To determine the frequencies of the genetic variants detected in the 5′-flanking region and the coding regions of the *CYP3A4* gene through sequence analysis, an additional 14 individuals from the Khoisan population and 50 individuals each from the Xhosa and MA populations were genotyped using a combination of restriction fragment length polymorphism (RFLP) analysis and additional sequence analyses. In cases where no restriction sites were created or destroyed by the SNPs of interest, mutagenic primers were designed to introduce artificial restriction enzyme recognition sequences (refer to Table 2 for the primer sequences used for RFLP genotyping). Amplification using the mutagenic primers was performed by means of a nested PCR, using 1 in 200 dilutions of PCR product as template, to avoid co-amplification of isoforms and pseudogenes. The nested PCR conditions were identical to those used during original PCR amplification, except that the cycle number and MgCl_2_ concentration were changed to 25 cycles and 2 mM, respectively (refer to Table 2 for annealing temperatures). To ensure that the RFLP assays were successful, samples with known genotypes were selected as controls for each of the individual restriction enzyme analyses. Due to the large number of variants detected in the exon 7 amplicon, all the individuals from all three of the population groups were sequenced for this region, rather than utilizing individual RFLP genotyping assays. Additionally, due to the fact that the RFLP genotyping assay for rs57409622 in exon 6 would detect both the presence of this SNP and the adjacent rs4986907 (allele defining SNP of CYP3A4^*^15), any individuals testing positive for this assay underwent bi-directional sequencing to determine which one, or both, of the SNPs were in fact present. For genotyping specifications, including a list of the specific restriction enzymes used, refer to Table 2.

**Table 2:** **PCR_RFLP primers, PCR conditions, and genotyping assays**.

**Position in gene**	**Allele**	**rs number**	**Genotyping assay**	**Primer name**	**Sequence (5′-3′)**	***T*_*m*_ (°C)**	**Product size (bp)**
−392 A>G	CYP3A4^*^1B	rs2740574	*Mbo*II	5′_F_m1	GGA CAG CCA TAG AGA CAA GGG **g**A	55	350
				5′_R	GGC TAT GTG CAT GGA GCT TT		
−292 T>G		Novel	*Rsa*I	5′_F	CAG AAG GGA TGA CAT GCA GA	55	575
				5′_R_m	CCT CCT TTG AGT TCA TAT TCT ATG AGG TAT C**g**T		
−215 T>A		rs144721069	*Hpy188*III	5′_F_m2	TGT GTG TGT GAT TCT TTG CCA ACT TC**t c**AG G	55	181
				5′_R	GGC TAT GTG CAT GGA GCT TT		
3847 A>G		Novel	*Nla*III	E2_F_m	GCA GGA AAG GAC CTG ATG AAC A**c**A T	55	323
			E2_R	AAG CTG CTC TTG GCA ATC AT			
5916 T>C		rs12721625	*Bse*NI	E3_F	TGA CGT CTC CAA ATA AGC TTC C	60	301
				E3_R	AGG TTG ACA AGA GCT TCA TCC		
13969 G>A		Novel	*Apo*I	E5_F	TCT AGC ATA GGG CCC ATC AC	60	352
				E5_R	CA GTG GAC TAC CCC TTG GAA		
14268 C>T	CYP3A4^*^23	rs57409622	*Aci*I/Sequencing	E6_F_m/E6_F	GAT GTG TTG GTG AGA AAT CTG AG**a** C	55	362/154
				E6_R	GGA ATA ACC CAA CAG CAG GA		
14269 G>A	CYP3A4^*^15	rs4986907	*Aci*I/Sequencing	E6_F_m/E6_F	GAT GTG TTG GTG AGA AAT CTG AG**a** C	55	362/154
				E6_R	GGA ATA ACC CAA CAG CAG GA		
15619 A>G		rs111768354	Sequencing				
15628 C>T		rs4987159	Sequencing				
15649 A>T	CYP3A4^*^24	rs113667357	Sequencing				
15753 T>G		rs2687116	Sequencing				
15783 T>C		rs4987160	Sequencing				
15804 T>G		rs28988584	Sequencing				
15837 T>A		rs12721622	Sequencing				
17829 T>C		Novel	*Psi*I	E9_F	CAT CCT GCT TTC CAA GGA	60	418
				E9_R	CCT GCA TGC CTC TAG AAA GTG		
20230 G>A	CYP3A4^*^1G	rs2242480	*Rsa*I	E10_F	TGA TGC CCT ACA TTG ATC TGA	60	391
				E10_R	CTG CCA GTA GCA ACC ATT TG		
20309 G>C		rs4986911	*Hind*III	E10_F	TGA TGC CCT ACA TTG ATC TGA	55	327
				E10_R_m	CAG TGA AAG AAT CAG TGA TTA **a**G		
20327 T>C		rs34738177	*Mbo*II	E10_F	TGA TGC CCT ACA TTG ATC TGA	60	391
				E10_R	CTG CCA GTA GCA ACC ATT TG		
21896 C>T	CYP3A4^*^12	rs12721629	*Pst*I	E11_F	CCC GAA TGC TTC CCA CCT	55	334
				E11_R_m	CAT CTT TTT TGC AGA CCC TCT **gc**A		
23081 C>T		rs12721620	*HpyCH4*IV	E12_F	GAC TGA AAG CTC CTA TAG TGT C	60	598
				E12_R	CCA TGC TAA TCT ACA TGG GCT		
25721 A>G		rs3735451	*BsaJ*I	E13_F	GCC ATC ATA CCT AAT AAT CTG G	60 Xhosa	988
				E13_R	AT GTG CAG GAA AGC ATC TGA	55 Others	

5′, 5′-upstream; E, exon; F, forward; R, reverse; m, mutagenic primer; lowercase and bold letters, mutagenic bases for the introduction of restriction enzyme recognition sites.

### Statistical analysis

Allele frequencies of the *CYP3A4* genetic variants detected in the three population groups were compared using MedCalc Version 12.3.0 (http://www.medcalc.org/calc/odds_ratio.php). Furthermore, we compared the frequencies of the allele defining SNPs detected in the three South African populations to the frequencies reported by the 1000 Genomes Browser (http://browser.1000genomes.org/) and HapMap Phase I + II Project data (http://hapmap.ncbi.nlm.nih.gov/). The 1000 Genomes Browser contains allele frequency information for the African (AFR), American Admixed (AMR), East Asian (ASN) and European (EUR) populations; while the HapMap project contains frequency data for European (CEU), Chinese (CHB), Japanese (JPT) and Nigerian (YRI) populations. Deviations from Hardy-Weinberg equilibrium (HWE) were determined using either a Pearson chi-squared analysis or an analogue to Fisher's exact test, depending on observed genotype distribution, in Tools for Population Genetic Analysis (TFPGA) Software v1.3 (http://www.marksgeneticsoftware.net/tfpga.htm). *P*-values of <0.05 were considered statistically significant.

## Results

### Variant detection

This study detected 24 SNPs in 45 individuals from three South African populations using *CYP3A4* DNA sequencing. Three of the intronic SNPs and one SNP in the 5′-flanking region are novel. Genotyping of rs12721624 in intron 8 and rs147972695 in intron 12 could not be performed in the entire cohort, due to technical difficulties. Genotyping of the remaining 22 SNPs was successful, and all SNPs were in HWE (refer to Table 3 for the positions and frequencies of the detected SNPs). The previously described alleles CYP3A4^*^1B and ^*^1G were present in all three populations, while CYP3A4^*^12 and CYP3A4^*^15 were only present in the Xhosa population. Furthermore, two novel alleles, designated CYP3A4^*^23 and CYP3A4^*^24, which are characterized by the two non-synonymous SNPs, resulting in p.R162W and p.Q200H, were detected. CYP3A4^*^24 was present in all three population groups, while CYP3A4^*^23 was present in the Xhosa and Khoisan populations. Of particular interest, the amino acid change caused by R162W (CYP3A4^*^23) was predicted by both the SIFT and PolyPhen algorithms to affect the function of the protein product. None of the variants were predicted to alter any splice-sites.

**Table 3:** ***CYP3A4* variants detected in the three South African populations**.

**Position in gene**	**Allele**	**rs number**	**Region**	**Amino acid substitution**	**Allele frequencies (%)**		
**(ENSG00000160868)**					**Khoisan (*n* = 29)**	**Xhosa (*n* = 65)**	**MA (*n* = 65)**
−392 A>GG>A	CYP3A4^*^1B	rs2740574	5′-flanking		76.79	73.02	45.90
−292 T>G		Novel	5′-flanking		7.14	0.00	0.81
−215 T>A		rs144721069	5′-flanking		0.00	0.81	0.85
3847 A>G		Novel	Intron 1		0.00	8.59	0.00
5916 T>C		rs12721625	Intron 2		0.00	1.56	1.56
13969 G>A		Novel	Intron 5		0.00	0.00	2.31
14268 C>T	CYP3A4^*^23	rs57409622	Exon 6	R162W	3.57	0.77	0.00
14269 G>A	CYP3A4^*^15	rs4986907	Exon 6	R162Q	0.00	2.38	0.00
15619 A>G		rs111768354	Exon 7	G190G	1.72	3.85	3.17
15628 C>T		rs4987159	Exon 7	I193I	0.00	4.62	3.17
15649 A>T	CYP3A4^*^24	rs113667357	Exon 7	Q200H	10.34	3.08	3.17
15753 T>G		rs2687116	Intron 7		75.86	77.69	45.38
15783 T>C		rs4987160	Intron 7		10.34	3.85	3.85
15804 T>G		rs28988584	Intron 7		0.00	3.85	2.38
15837 T>A		rs12721622	Intron 7		10.34	10.00	3.85
17024 C>T^*^		rs12721624	Intron 8		0.00	0.00	3.33
17829 T>C		Novel	Intron 9		8.62	0.78	0.78
20230 G>A	CYP3A4^*^1G	rs2242480	Intron 10		91.38	93.85	60.00
20309 G>C		rs4986911	Intron 10		15.52	9.68	6.35
20327 T>C		rs34738177	Intron 10		0.00	1.61	0.78
21896 C>T	CYP3A4^*^12	rs12721629	Exon 11	L373F	0.00	2.34	0.00
23081 C>T		rs12721620	Intron 11		1.92	20.31	10.83
25721 A>G		rs3735451	Intron 12		76.92	87.70	50.00
25739 C>T^*^		rs147972695	Intron 12		0.00	3.33	0.00

Allele frequencies are given for the variant allele.

* Due to RFLP genotyping failure, these SNPs were only genotyped in the 45 sequenced individuals.

### Population variant frequency comparisons

When examining the successfully genotyped variants in the three South African populations, we noticed that the allele frequencies for several SNPs differed significantly between the population groups (*P* < 0.05) (refer to Table 4). The smallest difference was seen when the allele frequencies of the Khoisan and Xhosa populations were compared, with the allele frequencies of three SNPs differing significantly between these two population groups. With regards to the comparisons of (1) the Khoisan and MA populations, and (2) the Xhosa and MA populations, five and six SNPs showed significant allele frequency differences, respectively.

**Table 4:** **All allele defining variants and SNPs displaying significant differences in allele frequencies between the Khoisan (*n* = 29), Mixed Ancestry (*n* = 65), and Xhosa (*n* = 65)**.

**Position in gene**	**Allele**	**rs number**	**Khoisan-mixed ancestry**		**Khoisan-xhosa**		**Mixed ancestry-xhosa**	
**GenBank: AF280107.1**			***P*-Value**	**OR (95% CI)**	***P*-Value**	**OR (95% CI)**	***P*-Value**	**OR (95% CI)**
−392 A>GG>A	CYP3A4^*^1B	rs2740574	0.000	0.257 (0.125–0.525)	0.592	0.818 (0.392–1.705)	0.000	3.189 (1.876–5.421)
−292 T>G		Novel	0.619	1.769 (0.187–16.713)	0.041	21.343 (1.129–403.534)	0.093	15.894 (0.628–402.140)
3847 A>G		Novel	0.713	2.094 (0.041–106.852)	0.094	0.087 (0.005–1.508)	0.029	0.042 (0.002–0.716)
14268 C>T	CYP3A4^*^23	rs57409622	0.137	10.138 (0.478–214.853)	0.206	4.778 (0.424–53.807)	0.566	0.391 (0.016–9.687)
14269 G>A	CYP3A4^*^15	rs4986907	0.713	2.094 (0.041–106.852)	0.430	0.302 (0.015–5.935)	0.202	0.144 (0.007–2.818)
15649 A>T	CYP3A4^*^24	rs113667357	0.059	3.519 (0.953–12.993)	0.053	3.635 (0.985–13.414)	0.964	1.033 (0.253–4.2220)
15753 T>G		rs2687116	0.000	0.264 (0.132–0.529)	0.783	1.108 (0.534–2.298)	0.000	4.191 (2.447–7.180)
17829 T>C		Novel	0.025	11.981 (1.367–105.025)	0.025	11.981 (1.367–105.025)	1.000	1.000 (0.062–16.163)
20230 G>A	CYP3A4^*^1G	rs2242480	0.000	0.142 (0.053–0.378)	0.540	1.439 (0.450–4.603)	0.000	10.167 (4.583–22.553)
21896 C>T	CYP3A4^*^12	rs12721629	0.707	2.128 (0.042–108.589)	0.437	0.307 (0.016–6.030)	0.202	0.144 (0.008–2.817)
23081 C>T		rs12721620	0.083	0.161 (0.021–1.268)	0.013	0.077 (0.010–0.583)	0.043	0.477 (0.232–0.978)
25721 A>G		rs3735451	0.001	0.300 (0.143–0.629)	0.076	2.140 (0.922–4.965)	0.000	7.133 (3.718–13.685)

Variants showing significant differences between the South African populations are indicated in shaded areas (P < 0.05).

**Figure 1:**
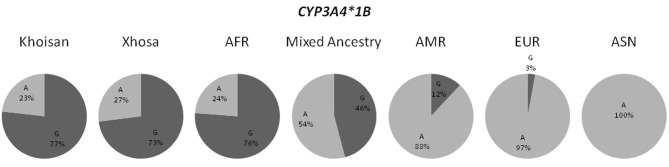
Distribution of the CYP3A4^*^1B allele frequencies in all three South African populations and the four populations present in the 1000 Genomes Browser. (AFR, African; AMR, American Admixed; ASN, East Asian; EUR, European).

The novel *CYP3A4* alleles, although detected in the South African populations (refer to Table 3), were present at frequencies of less than 1% in the populations recorded on the 1000 Genomes Browser. When examining the previously described *CYP3A4* alleles, in the case of CYP3A4^*^12 and CYP3A4^*^15, the data from the 1000 Genomes Browser was not available for all populations, therefore we utilized frequency data from the HapMap Project. Both of these variants were not detected in any of the HapMap populations, however, it should be noted that frequency data for the CYP3A4^*^15 SNP was not available for the YRI population. These SNPs were also absent in the MA and Khoisan populations, but were present in the Xhosa population at a frequency of at 2.3% and 2.4%, respectively. With regards to the remaining potentially functional *CYP3A4* allele, namely CYP3A4^*^1B, the frequencies of this variant in the three South African populations as well as those reported by the 1000 Genomes Browser differed substantially, as demonstrated by Figure 1.

## Discussion

### *CYP3A4* genetic variation in the three South African populations

To our knowledge, this was the first study in which the entire coding region of the *CYP3A4* gene was screened for common genetic variation in any Southern African population. This study identified a total of 24 variants in the three South African population groups, which included the discovery of four novel SNPs (i.e., ~17% of the total genetic variation). Overall this study revealed the presence of the previously described CYP3A4^*^1B, CYP3A4^*^1G, CYP3A4^*^12, and CYP3A4^*^15 alleles, in addition to two novel alleles, CYP3A4^*^23 and CYP3A4^*^24. The number of novel alleles reported here is in accordance with the number of novel alleles that we have detected previously through the re-sequencing of other *CYP* genes in South African populations (Drögemöller et al., 2010; Wright et al., 2010). Prior to this study, these novel *CYP3A4* alleles had not been recorded on the *CYP* allele database and were present at very low frequencies in the populations described on the 1000 Genomes Browser. Both were present in the Xhosa and Khoisan populations examined in this study and CYP3A4^*^24 was additionally detected in the MA population. The p.R162W amino acid change in exon 6, characterizing CYP3A4^*^23, may have functional consequences for the CYP3A4 protein, as arginine is a positively charged and hydrophilic amino acid; while tryptophan is a polar, aromatic and hydrophobic amino acid. The likely functional consequences of this variant are in agreement with the predictions made by both the SIFT and PolyPhen algorithms. Although the p.Q200H variant, characterizing CYP3A4^*^24, was not predicted to change the function of the protein product, the presence of this variant has also been reported in the genome of another Khoisan individual sequenced by Schuster et al. (2010), which correlates to the fact that the frequencies of both novel alleles were highest in the Khoisan population (Drögemöller et al., 2011). Additionally, the low frequency of these variants in the 1000 Genomes populations in comparison to the presence of these alleles at varying frequencies in the South African population groups, highlights the unique genomic composition of South African populations. Thus, results obtained from other population groups cannot be directly inferred onto the South African populations and comprehensive re-sequencing studies such as this one are required to characterize South African genomes.

The recent discovery of the CYP3A4^*^22 allele confirmed that novel alleles may have functional relevance to the field of pharmacogenetics (Wang et al., 2011). This allele was initially found to influence RNA expression and statin dose requirement (Wang et al., 2011). These findings have subsequently been replicated with regards to statin therapy and the allele has additionally been shown to influence the dose requirements of the immunosuppressants, tacrolimus, and cyclosporine (Elens et al., 2011, 2012). CYP3A4^*^22 is characterized by the intron 6 SNP rs35599367, which, however, was not genotyped in the current study as the aim of the study was to examine only coding regions, including the exon-intron boundaries, and the core promoter region of the gene. Furthermore, this variant does not occur in the 1000 genomes AFR or ASN populations and occurs at very low frequencies (2–5%) in the EUR and AMR populations and is thus unlikely to occur at pharmacogenetically relevant frequencies in the South African populations (http://browser.1000genomes.org/Homo_sapiens/Variation/Population?r=7:9936581699366816;source=dbSNP;v=rs35599367;vdb=variation;vf=11936818). The reported functional significance of this intronic CYP3A4^*^22 variant does, however, highlight the importance of non-coding regions. The significance of these areas, including regions that are not in close proximity to the gene has been further emphasized by the recent release of the ENCODE data (ENCODE Project Consortium et al., 2012). These data suggest that in the future additional analyses to examine the variation present in such areas, including the functional significance of the four novel non-coding SNPs identified by this study, are warranted.

The detection of novel variants in this study highlights the fact that although large re-sequencing studies such as the 1000 Genomes Project have played an integral role in characterizing the human variome (1000 Genomes Consortium, 2010), novel variation still exists, underlining the importance of re-sequencing studies such as this one. These re-sequencing studies are specifically required in African populations, as these populations have been under-represented in genomic studies to date (Rosenberg et al., 2010; Drögemöller et al., 2011). Furthermore, it may be important to compare results obtained by next generation sequencing studies to those obtained through the use of Sanger sequencing. Although the throughput of next generation sequencing studies is beyond comparison, it may be beneficial to evaluate the accuracy of next generation sequencing for the complex and polymorphic *CYP* genes, whose sequences show high similarity to one another and to their corresponding pseudogenes (Drögemöller et al., 2011). This may be particularly important with regards to the genotyping of *CYP3A4*, which shows between 76 and 88% sequence similarity to the *CYP3A43*, *CYP3A5*, and *CYP3A7* genes (http://www.ensembl.org/Homo_sapiens/Gene/Compara_Paralog?g=ENSG00000160868;r=7:99354604-99381888) and is thus likely to be affected by the consequences of misalignment or co-amplification of other genes during the use of high-throughput technologies.

Of the previously identified alleles that were detected in this study, both CYP3A4^*^1B and CYP3A4^*^12 have been reported to have functional relevance for pharmacogenetic applications. The high frequency CYP3A4^*^1B is characterized by a 5′-upstream c.−392A>G point mutation in a regulatory element, namely the putative nifedipine-specific element, which has been linked to altered gene expression *in vitro* (Amirimani et al., 2003; Georgitsi et al., 2011). Furthermore, this allele has been associated with various disease states such as prostate cancer and secondary leukemias (Lamba et al., 2002). Of relevance to pharmacogenetic applications, PharmGKB lists this SNP as affecting the metabolism of a number of therapeutic drugs, although the level of evidence for variant-drug associations is still low currently (http://www.pharmgkb.org/rsid/rs2740574; Whirl-Carrillo et al., 2012). The lack of pharmacogenetic evidence for this allele is further questioned by the results obtained by Wang et al. (2011) and the functional significance of this variant may require further examination. On the other hand, while CYP3A4^*^1B appears to affect the expression of *CYP3A4*, CYP3A4^*^12 (p.L373F) affects the protein product. p.L373 has been identified as one of the key residues affecting substrate binding, cooperativity and regioselection of metabolism (Sevrioukova and Poulos, 2013) and therefore the amino acid change has been shown to result in a protein that amongst others, displays an altered testosterone metabolite profile and a four-fold increase in the *Km* value for 1′-OH midazolam formation (Eiselt et al., 2001). While CYP3A4^*^1B occurs at a relatively high frequency, both CYP3A4^*^12 and CYP3A4^*^15 occur at low frequencies, possibly limiting the relevance that these two variants may have for pharmacogenetic applications, especially when considering their absence from the HapMap populations. Similarly, the lack of applicability of the SNPs defining CYP3A4^*^3, CYP3A4^*^13, CYP3A4^*^17, and CYP3A4^*^18 to pharmacogenetics in the South African setting is also likely as they were not detected in this study or a previous study (Ikediobi et al., 2011). These conclusions should however, be made with caution, as the relatively frequent occurrence of rare variants in African populations (Tishkoff et al., 2009) cannot be ignored and the effect of such variants should possibly also be taken into account when considering the implementation of pharmacogenetics in the African context.

### Variant frequency differences between the three South African populations

When comparing the significant differences in allele frequencies between the three population groups, it was observed that the three groups differed significantly from one another for eight SNPs (refer to Table 4). These results reflect the unique genomic compositions of South African populations (Warnich et al., 2011) and indicate that the results of one South African population are not always representative of another South African population. When looking at the three populations independently, the Khoisan and Xhosa were shown to be the most similar to one another, while the differences observed between the Khoisan and MA and the Xhosa and MA were comparable. The fact that the MA population showed the greatest number of genetic differences may be explained by the large number of ancestry contributions, other than the Xhosa and Khoisan, that have been made to this population (Schlebusch et al., 2009; De Wit et al., 2010; Quintana-Murci et al., 2010; Warnich et al., 2011). The large degree of similarity observed between the Xhosa and Khoisan is to be expected and can be explained by the large ancestry contribution that the Khoisan have made to the Xhosa population (De Wit et al., 2010; Warnich et al., 2011).

The differences in allele frequencies observed for the CYP3A4^*^1 sub-allele, CYP3A4^*^1B, between the different population groups (refer to Figure 1), serves as an excellent illustration of how pharmacogenetic applications may differ between population groups. It is important to remember that drugs designed to optimally treat one population group based on the presence of a certain allele, may be harmful to another population group for which the opposite allele is dominant. Figure 1 shows how the CYP3A4^*^1B allele is more frequent in the African populations (Khoisan, Xhosa and AFR), while in the ASN and EUR population groups the opposite allele occurs more often. Interestingly, both the MA and AMR admixed populations show allele frequencies that are intermediate between the African and EUR/ASN populations. Furthermore, the MA is more similar to the African populations, while the AMR is more similar to the non-African populations. These results are in accordance with the ancestral history of these population groups. The MA have ancestry contributions from the Xhosa, Khoisan, European, and Asian populations (Schlebusch et al., 2009; Tishkoff et al., 2009; De Wit et al., 2010; Quintana-Murci et al., 2010), which explains why although the frequencies of the variants in this population are between the African and EUR/ASN populations, they are more similar to the African populations. On the other hand, the AMR population, which consists of the Mexicans, Puerto Ricans, Columbians, and Peruvians (http://www.1000genomes.org/faq/which-populations-are-part-your-study), is more similar to the EUR/ASN population groups due to the larger ancestry contribution that these populations have made to the AMR, when compared to the ancestry contribution of Africans (Galanter et al., 2012).

The allele frequency differences observed in admixed populations, as previously reported in admixed Brazilian populations (Suarez-Kurtz, 2005, 2010; Suarez-Kurtz et al., 2012), bring to light an important consideration for the implementation of pharmacogenetics. Individuals within admixed populations are likely to exhibit different levels of ancestry contributions, as has been shown with the use of STRUCTURE analyses for both the MA (Tishkoff et al., 2009) and Brazilian populations (Suarez-Kurtz, 2010). Population based pharmacogenetic testing is thus unlikely to detect all pharmacogenetically relevant variants and it may be necessary to implement pharmacogenetics on an individualized level. In the context of South Africa with its diverse population groups, which exhibit both rare variants and variants from several different population sources (Warnich et al., 2011), this may be especially important. However, before this goal can be realized it will be necessary to consider whether individualized treatment will be feasible in the resource limited settings of the country.

## Conclusions

Although this study identified both novel and known SNPs of functional significance in all three population groups, due to the current lack of validated evidence regarding the pharmacogenetic application of *CYP3A4*, the relevance of these SNPs in the clinical setting remains unknown. The SNP markers detected in the current study should therefore be included in genotyping panels in future pharmacogenetic association studies involving CYP3A4 substrate medications. Nonetheless, this study provides an excellent example of how re-sequencing studies are required in African populations in order to identify variation that remains novel. These differences in allele frequencies were not only seen when comparing the South African populations to other populations, but also when comparing them to each other. These results demonstrate that a one-size-fits-all approach is not ideal when implementing therapeutic treatment regimes, also within the South African context.

### Conflict of interest statement

The authors declare that the research was conducted in the absence of any commercial or financial relationships that could be construed as a potential conflict of interest.
